# Psychometric testing of the Iranian version of the TeamSTEPPS teamwork perception questionnaire: a cross-cultural validation study

**DOI:** 10.1186/s12913-021-06739-z

**Published:** 2021-07-17

**Authors:** Edris Kakemam, Mahtab Rouzbahani, Mohammad Reza Rajabi, Young Sook Roh

**Affiliations:** 1 Department of Health Services Management, School of Management and Medical Informatics, Tabriz University of Medical Sciences, Tabriz, Iran; 2 Tabriz Health Services Management Research Center, Tabriz University of Medical Sciences, Tabriz, Iran; 3 Department of Health Management and Economics, School of Public Health, Tehran University of Medical Sciences, Tehran, Iran; 4 Department Cardiology, Faculty of Medicine, Shahed University, Tehran, Iran; 5 Chung-Ang University, Red Cross College of Nursing, Seoul, Republic of Korea

**Keywords:** Perception, Confirmatory factor analysis, Nurse, Reliability, Teamwork, patient safety

## Abstract

**Background:**

The use of validated questionnaires to assess the perception of teamwork can be an early step in improving team training activities. Team-STEPPS® Teamwork Perception Questionnaire (T-TPQ) has been adapted and validated for hospital setting use in several countries. Due to linguistic and cultural differences, there is need to test the psychometrics of the adapted versions. However, no research have not yet assessed the psychometric properties of the Persian T-TPQ. Therefore, this study aims to assess the internal consistency reliability and construct validity of an Iranian version of the Team-STEPPS® Teamwork Perception Questionnaire (IR-T-TPQ).

**Methods:**

To conduct this study, we undertook a cross-sectional survey approach between May 2020 and January 2021. In total, 404 nurses were recruited by convenience sampling technique from 10 teaching hospitals in Tabriz, Iran. Internal consistency reliability was analyzed using Cronbach’s alpha coefficient. Confirmatory factor analysis was performed to test the construct validity of the instrument.

**Results:**

Cronbach’s alpha coefficients for each subscale were acceptable, ranging from 0.84 to 0.92, as well as for the total IR-T-TPQ (α = 0.96). The confirmatory factor analysis demonstrated a five-factor model, all of whose fit indices were acceptable, except for the goodness-of-fit index and normed fit index (χ2 (df) 1332 (550), *p* < 0.001, Normed chi-square (χ2/df) = 2.423, RMSEA = 0.059, TLI = 0.897, CFI = 0.904, AGFI = 0.814).

**Conclusions:**

The psychometric properties of the IR-T-TPQ resulted in acceptable levels of internal consistency reliability and construct validity, respectively, in Iranian hospital nurses. Further study is needed to compare the teamwork level of nurses in various settings or to evaluate the effectiveness of the teamwork intervention using this validated and reliable tool.

## Introduction

Since the release of the Institute of Medicine (IOM) report, To Err Is Human, in 2000, the importance of teamwork to warrant patient care safety and health care quality in health care settings has been emphasized internationally [1]. Teamwork is defined as a dynamic process involving two or more health care professionals with complementary backgrounds and skills, sharing common health goals and exercising concerted physical and mental effort in assessing, planning, or evaluating patient care [2]. The nursing unit maintains the continuity of work 24 h a day through three shifts and cooperatively performs a series of nursing care activities (attends to patient safety through handover). Educators and administrators should assess the teamwork level and adopt teamwork training for nurses because nurses play a key role in patient care safety and quality of health services. One challenge of team research has been to develop reliable and relevant assessment tools for measuring the development and performance outcomes of training programs. The Team Strategies and Tools to Enhance Performance and Patient Safety (TeamSTEPPS®), developed by the U.S. Department of Defense (DOD) and the Agency for Healthcare Research and Quality (AHRQ) [3], is an evidence-based program contribute to improving quality and safety in health care. All these strategies and tools lead to promoting communication, reducing medical errors or adverse events, and improving patient satisfaction and outcomes [4].

Although there are many questionnaires to measure teamwork among health care professionals, however, validity and reliability have not been confirmed for most of these tools [5, 6]. The TeamSTEPPS® Team Perception Questionnaire (T-TPQ) was developed to measure an individual’s perception and attitude of the group- or unit-level teamwork knowledge, skills and behaviors [7]. The T-TPQ is a self-report questionnaire comprising 35 items that measuring five subscales Team Structure Leadership, Situation Monitoring, Mutual Support, and Communication. Each subscales is covered by seven items [7].

The validated T-TPQ can be applied to understand the impacts of team training, as it is administrated before and after the training [8]. To date, the T-TPQ tool has been translated and validated among Korean nurses [9], Norwegian [10, 11], Greek [12], Swedish [13] and Japanese healthcare professionals [14], and Chinese residents [15], showing acceptable reliability and validity. All versions of the T-TPQ contp <ains the same content, with minor modifications to reflect the clinical practices.

Teamwork is essential for patient safety and is suggested as an effective strategy for promoting the treatment process of patients [16]. Perceived teamwork is associated with the better patient care safety and health care quality [17], lower adverse events [9, 18], higher job satisfaction [1, 19], better job performance [20], and higher patient outcomes [19]. Therefore, considering the importance and impact of the perceived teamwork, a tool with good psychometric properties is pertinent to assess the level of teamwork of nurses accurately.

Researchers need a reliable, valid, and culturally adapted tool to assess perceived teamwork in their own context as a predictor for patient safety or an outcome variable after an intervention. The assessment results of the perceived level of teamwork using a valid and reliable survey can be used as basic data for the development and assessment of education or training for improving the patient care safety and health care quality. Additionally, to generalize the results of research using the T-TPQ, it is necessary to conduct a validation study to evaluate the relevance of the tool for health care professionals in other countries. Although many studies have validated the T-TPQ among healthcare professionals and physicians, there are few studies on the T-TPQ validation for nurses. In addition, due to linguistic and cultural differences, there is need to test the psychometrics of the adapted versions. Despite the importance of teamwork measurement, there is no available teamwork measurement tool in Iran so far. Moreover no research have not assessed the psychometric properties of the Persian T-TPQ. Therefore, the present study aimed to test the reliability and construct validity of the Iranian versions of the TeamSTEPPS Teamwork perceptions questionnaire (IR-T-TPQ).

## Methods

### Study design

To conduct this study, we performed a cross-sectional survey design to test the reliability and construct validity of the IR-T-TPQ. The research is reported based on the Strengthening the Reporting of Observational Studies in Epidemiology (STROBE) checklist for cross-sectional studies.

### Setting and participants

The study was carried out at 10 teaching hospitals in Tabriz, Iran. There are 20 hospitals in Tabriz to provide medical services, 10 of which are affiliated with the government and the Ministry of Health. In addition to providing medical services, these hospitals also provide educational services to students. They operate as referral hospitals in the northwest of the country and patients from neighboring provinces come to receive specialized services. Given that, the organizational structure and management of public and teaching hospitals are the same in all public hospitals in Iran and are managed under the same rules and regulations by Ministry of health, these hospitals can be partly considered as representative for the entire hospitals system of Iran. Out of 10 selected hospitals, three hospitals were large hospitals (> 300 beds), five medium hospitals (100–300 beds) and two small hospitals (less than 100 beds).

The target population included all nurses working in the mentioned hospitals. Inclusion criteria were nurses, defined as full-time nurses with a minimum of 1 year of experience with the current hospital and holding an at least Bachelor degree in Nursing. New recruitment nurses and part-time nurses were excluded from the study due to lack of familiarity with the climate of hospital and teams. In order to ensure at least 11:1 participant to items ratio, the sample size was set to a minimum of 385 participants [21]. To account for a non-response rate of 15%, a sample size of 442 was requested from the participating hospitals. Therefore, we selected a total of 442 nurses working in 10 hospitals using a convenience sampling method. Each hospital was assigned a proportional quota based on the nursing staff, and a proportional allocation was used to give the required sample size from that hospital to the various nursing wards. Of 442 distributed questionnaires, 410 (response rate: 92.8%) were returned, and 404 were analyzed after excluding sex incomplete questionnaires. Of the 21 nurses who did not returned the questionnaires, 10 nurses withdrew from the study, and the rest of the nurses did not complete the survey due to loss of questionnaires.

### Measures

The T-TPQ contains 35 survey items measuring five subscales: Team Structure, Leadership, Situation Monitoring, Mutual Support, and Communication. Each subscale contains seven items measured on a five-point Likert scale (1 = strongly disagree to 5 = strongly agree). The translation process followed a stepwise back-translation model for cross-cultural research in a process described in the following five steps [22]:

1. Forward-translation: the T-TPQ was translated into Persian by a professional bilingual translator with Persian as their native language;
2. Reviewing: to obtain cross-cultural validity, the translated version of the T-TPQ was reviewed by (a) a group of three nurses with expert knowledge in the field of teamwork in collaboration with the members of the research team, and (b) five nurses with experience from clinical practice to help confirm the cultural relevance of the T-TPQ in the context of Iranian hospital settings. This step generated some semantic and conceptual changes and resulted in a preliminary-translated version;
3. Back-translation: the T-TPQ was back-translated by a second professional bilingual translator with English as their native language, who was blinded to the original English version;
4. Comparison: members of the research team compared the back-translated version and the original version. In this step, only minor inconsistencies were discovered, thereby resulting in some minor revisions;
5. Pilot testing: to strengthen both the semantic and the content equivalence [23], the translated version was given to 15 nurses recruited from a single hospital. They were asked to comment on items they found unclear [24] and respond on a scale from 1 to 5 as to whether the items in the questionnaire were relevant, precise, understandable, and well-articulated. The pilot testing produced some semantic and conceptual changes and resulted in a final translated Persian version. Totally, minor changes were made in expressions to favour semantic equivalence, and the wording was adapted to the field of nursing studies (for example, the term healthcare professional was changed to nurse or the term manager was changed to head nurse).

### Data collection

The authors contacted the managers of hospitals and obtained permission to distribute the questionnaires. These two authors went around the units and distributed the paper-version questionnaires directly to the nurses according to the quota for the hospital. Due to the heavy workload of nurses, the researchers left behind the questionnaires with the staff nurses to complete. They asked the nurses to deliver the completed questionnaire to the unit office. A cover letter inviting participation in the current study attached to the questionnaires. The cover letter outlined the objectives of the research and briefly highlighted the topic to improve the response rate. Additional actions that were taken in order to improve the response rate during the study period, they several times visited the units to collect the questionnaires. During the collection, the researchers carefully assessed each questionnaire to ensure that it had been fully completed. The survey was conducted between May 2020 and January 2021.

### Ethical considerations

First, we obtained ethical approval of the study protocol from the ethics committee of Tabriz University of Medical Sciences (IR.TBZMED.REC.1397.1079). Permission to conduct the study was obtained from the managers at each hospital. Before data collection, verbal informed consent was obtained from each participant after a thorough explanation of how to complete the questionnaires and study goals. Anonymity and confidentiality were maintained by respecting and protecting each respondent’s rights not to disclose any of the information divulged to the researchers. Moreover, the participation of nurses in the study was completely voluntary and they were free to withdraw from the study whenever they wished.

### Data analysis

We analyzed the data using SPSS 23.0 (IBM Corp., Armonk, NY, USA) and IBM AMOS version 21.0 (IBM Corp.). Internal consistency (measured by Cronbach’s alpha) was assessed for the total questionnaire and each teamwork subscale and interpreted as acceptable when Cronbach’s alpha coefficient value was higher than 0.7 [15, 25]. Item analysis was calculated using the corrected item-total correlation and Cronbach’s alpha if an item was deleted. Pearson’s correlation coefficient (r) was used to analyze the correlations between each subscale of the T-TPQ. Construct validity was analyzed using the confirmatory factor analysis (CFA), which is very important for scales that have been culturally adapted [11, 13, 15]. The strength of the model was assessed using the chi-square goodness-of-fit (χ2), normed chi-square (χ2/df), root mean square error of approximation (RMSEA), Tucker-Lewis index (TLI), comparative fit index (CFI), goodness-of-fit index (GFI), normed fit index (NFI), adjusted goodness-of-fit index (AGFI), and the standardized root mean square residual (SRMR). It is interpreted as acceptable when normed χ2 < 3, RMSEA < 0.08, CFI > 0.90, GFI > 0.85, AGFI > 0.85, and NFI > 0.90. The χ2 should have a *p*-value of > 0.05 [26–29].

## Results

### Characteristics of sample

In total, 404 nurses completed the IR-T-TPQ and returned them to the researchers (response rate, 92.8%). The demographic characteristics of the participants are presented in Table 1. The respondents had an average age and work experience in nursing of 34.4 (SD = 8.3) and 10.8 years (SD = 8.1), respectively.

**Table 1:** Characteristics of the nurses (*n* = 404)

Variable	Frequency	Percentage (%)
**Gender**		
Male	101	25.0
Female	303	75.0
**Age (years)**		
23–30	173	42.8
31–40	133	32.9
> 40	98	24.3
**Education**		
Bachelor’s degree	361	89.4
Master’s or PhD degree	43	10.6
**Work experience in nursing (year)**		
≤ 5	140	34.7
6–10	101	25.0
> 10	163	40.3
**Job position**		
Staff nurse	379	93.8
Manager	25	6.2
**Workplace**		
General wards	269	66.6
Intensive care units	73	18.1
Emergency department	62	15.3

### Reliability

The results of the corrected item-total correlation, and Cronbach’s alpha for each subscale and if the item was deleted is presented in Table 2. The corrected item–total correlations of the 35-item scale ranged from 0.51 to 0.80 and can be considered acceptable because at least 50% of the retained items had total scores in the range of 0.30–0.70 [30]. The corrected item-total correlations were above 0.30 for items in all subscales. Cronbach’s alpha was 0.96 for the total IR-T-TPQ and ranged from 0.84 (Mutual Support) to 0.92 (Leadership) for the five subscales. Also, the mean scores and standard deviations for the five teamwork dimensions and the items are displayed in Table 2.

**Table 2:** Summary of reliability and mean scores and standard deviations for IR-T-TPQ items and subscales (*n* = 404)

Item (No. of items)	Corrected Item-Total Correlation	Cronbach’s Alpha if Item Deleted	Cronbach’s Alpha	Mean (SD)
**Team Structure (7)**			**0.85**	**3.85 (0.66)**
The skills of nurses overlap sufficiently so that the work can be shared when necessary.	0.51	0.85		3.78 (0.91)
Nurses are held accountable for their actions.	0.54	0.84		4.14 (0.83)
Nurses within my unit share information that enables timely decision making by the patient care team.	0.63	0.83		3.91 (0.84)
My unit makes efficient use of resources (e.g., staff, supplies, equipment, and information).	0.62	0.83		3.68 (1.02)
Nurses understand their roles and responsibilities.	0.67	0.83		3.82 (0.86)
My unit has clearly articulated goals.	0.67	0.82		3.79 (0.94)
My unit operates at a high level of efficiency.	0.67	0.82		3.83 (0.92)
**Team Leadership (7)**			**0.92**	**3.73** (0**.87**)
My head nurse considers nurses input when making decisions about patient care.	0.77	0.91		3.85 (1.03)
My head nurse provides opportunities to discuss the unit’s performance after an event.	0.76	0.91		3.77 (1.01)
My head nurse takes time to meet with nurses to develop a plan for patient care.	0.77	0.91		3.65 (1.09)
My head nurse ensures that adequate resources (e.g., staff, supplies, equipment, and information) are available.	0.72	0.92		3.68 (1.06)
My head nurse resolves conflicts successfully.	0.76	0.91		3.62 (1.12)
My head nurse models appropriate team behavior.	0.80	0.91		3.75 (1.04)
My head nurse ensures that nurses are aware of situations or changes that may affect patient care.	0.76	0.91		3.79 (0.99)
**Situational Monitoring (7)**			**0.87**	**3.64** (**0.71**)
Nurses effectively anticipate each other’s needs.	0.63	0.85		3.54 (0.96)
Nurses monitor each other’s performance.	0.56	0.86		3.68 (0.93)
Nurses exchange relevant information as it becomes available.	0.70	0.85		3.71 (0.92)
Nurses continuously scan the environment for important information.	0.70	0.84		3.67 (0.90)
Nurses share information regarding potential complications (e.g., patient changes, bed availability).	0.64	0.85		3.84 (0.88)
Nurses meet to re-evaluate patient care goals when aspects of the situation have changed.	0.66	0.85		3.39 (1.09)
Nurses correct each other’s mistakes to ensure that procedures are followed properly.	0.65	0.85		3.68 (0.89)
**Mutual Support (7)**			**0.84**	**3.80** (**0.66**)
Nurses assist colleagues during high workload.	0.53	0.83		3.80 (1.03)
Nurses request assistance from colleagues when they feel overwhelmed.	0.53	0.82		3.88 (0.80)
Nurses caution each other about potentially dangerous situations.	0.65	0.81		3.94 (0.78)
Feedback between nurses is delivered in a way that promotes positive interactions and future change.	0.71	0.80		3.87 (0.85)
Nurses advocate for patients even when their opinions conflict with that of a senior member of the unit.	0.51	0.83		3.767 (0.96)
When nurses have a concern about patient safety, they challenge others until they are sure that the concern has been heard.	0.64	0.81		3.80 (0.92)
Nurses resolve their conflicts, even when the conflicts have become personal.	0.59	0.82		3.57 (1.07)
**Communication (7)**			**0.89**	**3.83** (**0.69**)
Information regarding patient care is explained to patients and their families in lay terms.	0.64	0.88		4.00 (0.77)
Nurses relay relevant information in a timely manner.	0.72	0.87		3.89 (0.87)
When communicating with patients, nurses allow enough time for questions.	0.73	0.87		3.78 (0.89)
Nurses use common terminology when communicating with each other.	0.68	0.87		3.86 (0.91)
Nurses verbally verify information that they receive from one another.	0.69	0.87		3.80 (0.85)
Nurses follow a standardized method of sharing information when handing over patients.	0.71	0.87		3.79 (0.89)
Nurses seek information from all available sources.	0.65	0.88		3.66 (1.05)
IR-T-TPQ -Total scale			**0.96**	3.77 (0.61)

### Construct validity

**Fig. 1:**
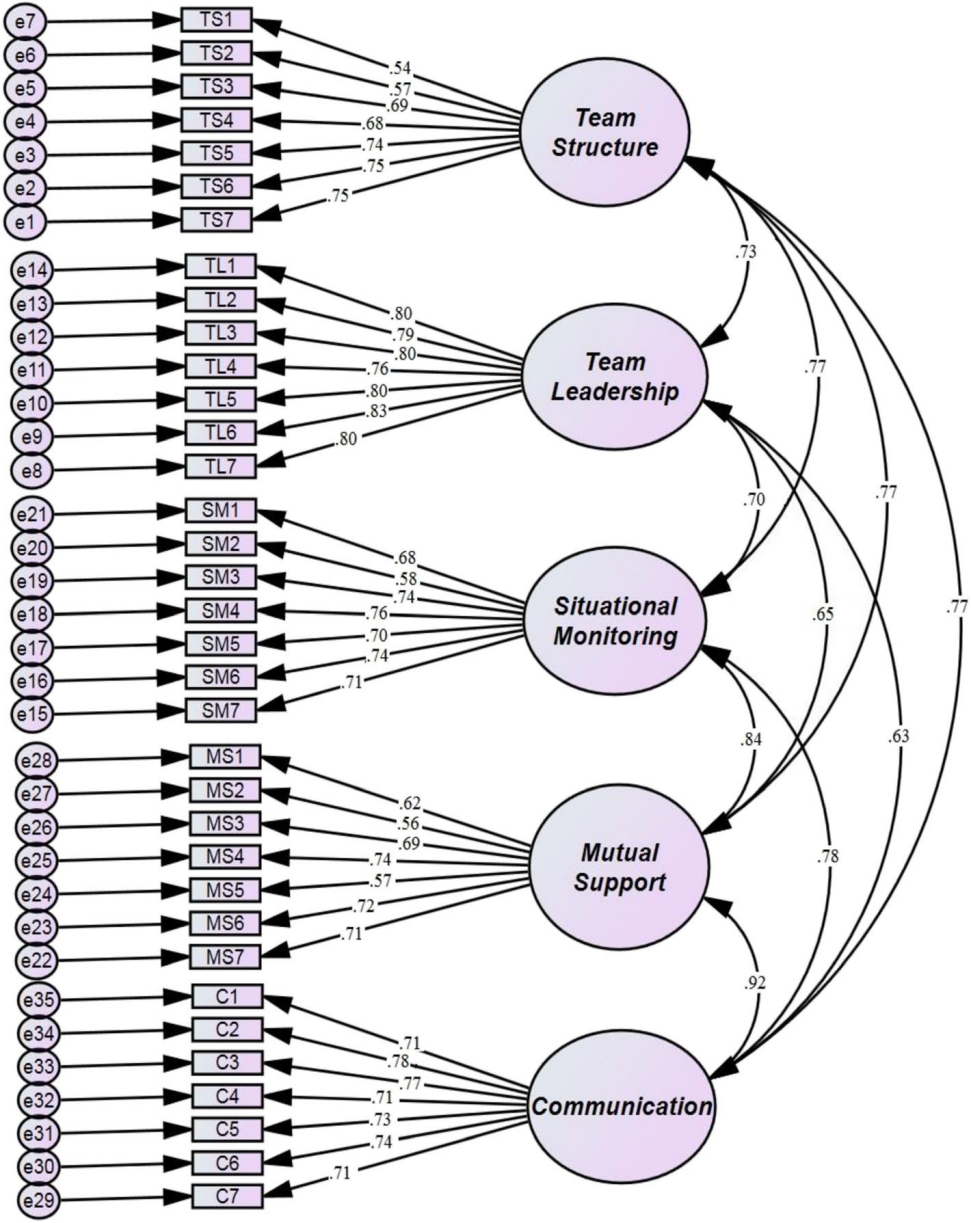
Confirmatory analysis model with factor loadings and correlations for the five IR-T-TPQ subscales

The analysis yielded a 35-item five-factor model that fit the data from the IR-T-TPQ very well (Fig. 1). The CFA demonstrates the fit indices of a five-factor model. The good-ness-of-fit indexes in the CFA revealed a χ2/df of 2.42, RMSEA of approximately 0.059, TLI of 0.897, and CFI of 0.904 (Table 3). Except for the GFI and NFI, the fit indices in our study were close to the cutoff criteria [26–29]. The factor loadings ranged from 0.54 to 0.83 for all items, and the correlations between the subscales were between 0.63 and 0.92. Therefore, using this rule, the five-factor structure was confirmed as resulting in a good model fit, thereby contributing to the stability of the tool.

**Table 3:** Confirmatory factor analysis fits for the five IR-T-TPQ subscales (*n* = 404)

Index	Index Criteria (***n*** > 250)	Fit Index in Iranian Sample (***n*** = 404)
*χ*^*2*^ (df), *p*-value	*p* < 0.05	1332 (550), *p* < 0.001
Normed chi-square (χ2/df)	< 3	2.423
RMSEA (CI)	< 0.08	0.059 (0.055, 0.063)
TLI	> 0.90	0.897
CFI	> 0.90	0.904
NFI	> 0.90	0.848
GFI	> 0.85	0.838
AGFI	> 0.85	0.814
SRMR	0.08	0.041

*RMSEA* Root mean square error of approximation, *CI* confidence interval, *TLI* Tucker-Lewis index, *CFI* Comparative fit index, *NFI*: Normed fit index, *GFI* Goodness-of-fit index, *AGFI* Adjusted goodness-of-fit index, *SRMR* Standardized root mean square residual

### Correlations among the subscales of the IR-T-TPQ

Table 4 shows the correlations (r) among the subscales of the IR-T-TPQ. The IR-T-TPQ indicated a significant correlation between each subscale of the questionnaire. The highest correlation coefficient was between Mutual Support and Commutation (r = 0.801; *p* < 0.001).

**Table 4:** Pearson correlations among the subscales of the IR-T-TPQ (*n* = 404)

	Team Structure	Leadership	Situation Monitoring	Mutual Support	Communication
Team Structure	–	0.654**	0.669**	0.653**	0.674**
Leadership		–	0.633*	0.581**	0.570**
Situation Monitoring			–	0.738**	0.688**
Mutual Support				–	0.801**

**p < 0.001

## Discussion

This study tested the internal consistency reliability and construct validity of the IR-T-TPQ among hospital nurses. The IR-T-TPQ was originally developed in the USA to serve as an alternative or complementary measure of teamwork behavior [29]. We compared the cross-cultural knowledge with the use of formally validated and established instruments [31]. This questionnaire can be used as an outcome indicator of TeamSTEPPS® or similar programs for improving team training [32]. In Iran, interprofessional teamwork has gained more focus in recent years, although no specialized programs, such as TeamSTEPPS®, have thus far been developed and implemented in healthcare organizations. IR-T-TPQ can help to the knowledge, attitude and skill of teamwork in Iranian nurses.

Our findings demonstrate the acceptable internal consistency reliability of the IR-T-TPQ. The five-factor IR-T-TPQ showed satisfactory internal consistency, with a total Cronbach’s alpha of 0.96, above the recognized threshold of 0.70. The result of reliability was in line with the prior validation studies of the T-TPQ in other countries, such as Korea [9], Norway [10, 11], China [15], the USA [32], and the original English version [8]. In contrast, in the Swedish study by Hall-Lord et al., Cronbach’s alpha values were low in Mutual Support and Communication [13]. Therefore, we can measure the perceived teamwork of nurses at their typical workplace with good internal consistency reliability. However, because reliability can be influenced by social desirability, as well as the main concept of the tool, it is necessary to supplement objective and multifaceted measures of teamwork that can overcome subjective measurement bias [29].

Our results showed satisfactory construct validity using CFA All the fit indices of the entire model were acceptable. Structural validity refers “to the extent to which the structure of a multi-item scale adequately reflects the hypothesized dimensionality of the construct being measured” [23] (p. 318). The CFA revealed that the original five- subscale structures of the T-TPQ provide a generally satisfactory fit for our study data, and the finding was consistent with the former validation studies of the T-TPQ [10, 15, 32]. The factor load of all items was acceptable. Our study exhibited better factor load than that in the study of the T-TPQ performed in Sweden [13] and China [15]. Overall, these results show that the model of the IR-T-TPQ is appropriate for future research in Iran.

The RMSEA was 0.059, and χ2/df was below 3. According to the recommendations for CFA, these goodness-of-fit indexes of CFA indicated an acceptable fit with the original construct [29], and the result was in agreement with the previous validation Chinese study of the T-TPQ (RMSEA = 0.059) [15]. Some previous studies that also reported acceptable RMSEA values include the Norwegian study by Ballangrud et al. (RMSEA = 0.069) [10], the Japanese study by Unoki et al. (RMSEA = 0.062) [14], and the USA study by Keebler et al. (RMSEA = 0.068) [32]. Furthermore, the Swedish study by Hall-Lord et al. [13] reported the RMSEA as 0.076. The original version of the T-TPQ has a rigorous structure, and the present Iranian version of the T-TPQ maintained its validity. The CFI (0.904) was slightly above the cut-off values for satisfactory evidence of model fit and was higher than in the previous validation studies [10, 13, 15] but lower than the CFI reported by Keebler et al. of 0.925 [32]. The TLI (0.897) was slightly below the recommended goodness-of-fit index (> 0.95) but higher than in the previous validation studies [11, 13, 15]. However, the studies considered the RMSEA the most robust and informative criterion in covariance structure modeling [10, 26, 27].

The correlations, conducted as a part of CFA, between the five subscales demonstrated significant correlations among the subscales of the IR-T-TPQ. Similar results have been reported in previous validation studies [10, 15, 32]. Findings obtained from our study illustrated that the correlation coefficient between Mutual Support and Commutation was the highest, indicating that if team members had better communication, the mutual sup-port of the team could be improved. The Norwegian study revealed that the highest correlation coefficient was between Team Structure and Communication [10]. However, the Chinese study showed that the correlation coefficient between Team Structure and Leadership was the highest [15]. Keebler et al. demonstrated that the Situation Monitoring strongly correlated with Mutual Support, thereby showing that the situation monitoring skill of health professionals could be enhanced by improving the mutual support [32].

The response rate was satisfactory (92.9%) compared with similar validation studies among health care personnel from Sweden (39.4%) [13], Norway (39.9%) [10], and among residents in China (83%) [15]. This discrepancy can be attributed to the study sample, which, in other studies, included health care personnel (nurses, physicians, midwife, occupational therapist, and physiotherapist). One of the important and fundamental factors in the CFA is the sample size. In support of this statement, Polit and Yang provided evidence that the larger sample size may have led to a better fit within the data [29]. In study conducted by Keebler et al. [32], the sample was 1700 employees from the U.S. Army medical facilities, which could explain the comparatively better outcome. Also, the sample size of the Norwegian and Swedish studies was 244 and 458, respectively [10, 13]. The sample size in another study was 664 Chinese residents [15]. In the current study, 404 participants provided 11 cases for each parameter, supporting the recommendations of at least 10 cases per item [21].

### Limitations

Both the strength and major contribution of this study is its establishment of satisfactory internal consistency reliability and construct validity in the IR-T-TPQ. We provided a Persian version of the T-TPQ, which may act as a basis for future studies on teamwork perception in the health care setting of Iran. However, the respondents of the research were from 10 teaching hospitals in Tabriz, Iran. For this reason, any generalization or interpretation of the results should be made with caution. The second limitation of our study was that we did not include the physicians in the survey. Usually, the proportion of physicians on other staff in the hospitals is lower. They refuse to participate in research because they are too busy.

## Conclusions

Our findings provided evidence that the IR-T-TPQ has the potential for measuring the teamwork perception of the Iranian nurses in hospital settings. Therefore, this scale can be used in teamwork training programs and research. Moreover, this questionnaire may help assess teamwork in hospital settings, which may facilitate improvement in the quality of care. Further cross-cultural comparative studies of the T-TPQ are required, with samples representing both health care professionals and nurses from various health care settings.

## Data Availability

The datasets used and analyzed during the current study are avail-able from the corresponding author upon request.

## Abbreviations

T-TPQ: Team-STEPPS® teamwork perception questionnaire
IR-T-TPQ: Iranian versions of the team-STEPPS® teamwork perception questionnaire
IOM: Institute of medicine
TeamSTEPPS®: Team strategies and tools to enhance performance and patient safety
DOP: Department of defense
AHRQ: Agency for healthcare research and quality
STROBE: Strengthening the reporting of observational studies in epidemiology
CFA: Confirmatory factor analysis
RMSEA: Root mean square error of approximation
TLI: Tucker-lewis index
CFI: Comparative fit index
GFI: Goodness-of-fit index
AGFI: Adjusted goodness-of-fit index
SRMR: Standardized root mean square residual
NFI: Normed fit index
CI: Confidence interval
